# Prognostic value of initial QRS analysis in anterior STEMI: Correlation with left ventricular systolic dysfunction, serum biomarkers, and cardiac outcomes

**DOI:** 10.1111/anec.12791

**Published:** 2020-08-26

**Authors:** Marta López‐Castillo, Álvaro Aceña, Ana M. Pello‐Lázaro, Vanessa Viegas, Beatriz Merchán Muñoz, Rocío Carda, Juan Franco‐Peláez, Maria Luisa Martín‐Mariscal, Sem Briongos‐Figuero, Jose Tuñón

**Affiliations:** ^1^ Department of Cardiology IIS‐Fundación Jiménez Díaz Madrid Spain; ^2^ Department of Urology Hospital La Princesa Madrid Spain; ^3^ Department of Hematology Hospital de Guadalajara Guadalajara Spain; ^4^ Department of Cardiology Hospital Rúber Juan Bravo Madrid Spain; ^5^ Department of Cardiology Hospital Infanta Leonor Madrid Spain

**Keywords:** electrocardiogram, myocardial infarction, Q waves, QRS complex, systolic dysfunction

## Abstract

**Background:**

The presence of pathologic Q waves on admission electrocardiogram (ECG) in patients with anterior ST‐elevated myocardial infarction (STEMI) has been related to adverse cardiac outcomes. Our study evaluates the prognostic value of QRS complex and Q waves in patients with STEMI undergoing percutaneous coronary intervention.

**Methods:**

We prospectively analyzed the specific characteristics of QRS complex and pathologic Q waves on admission and on discharge ECG in 144 patients hospitalized for anterior STEMI. We correlated these findings with the development of left ventricular systolic dysfunction (LVSD), appearance of heart failure (HF) or death during follow‐up, and levels of several biomarkers obtained 6 months after the index event.

**Results:**

Multivariate logistic regression analysis showed that QRS width (odds ratios [OR] 1.05, *p* = .001) on admission ECG and the sum of Q‐wave depth (OR 1.06, *p* = .002) on discharge ECG were independent predictors of LVSD development. Moreover, QRS width on admission ECG was related to an increased risk of HF or death (OR 1.03, *p* = .026). Regarding biomarkers, QRS width on admission ECG revealed a statistically significant relationship with the levels of NT‐pro‐BNP at 6 months (0.29, *p* = .004); the sum of Q‐wave depth (0.27, *p* = .012) and width (0.25, *p* = .021) on admission ECG was related to the higher levels of hs‐cTnI; the sum of the voltages in precordial leads both on admission ECG (−0.26, *p* = .011) and discharge ECG (0.24, *p* = .046) was related to the lower levels of parathormone.

**Conclusions:**

Assessment of QRS complex width and pathologic Q waves on admission and discharge ECGs aids in predicting long‐term prognosis in patients with STEMI.

## 1 INTRODUCTION

The electrocardiogram (ECG) is an important tool for managing patients with suspected myocardial infarction (MI). As it is simple, cost‐effective, and fast to use, great effort has been made to study its components for possible use in assessing the prognosis of patients with MI. The presence of pathologic Q waves on the first ECG usually predicts a poor prognosis, as this finding is related to myocardial necrosis (Thygesen et al., 2019) and cardiac mortality in patients undergoing fibrinolysis (Andrews, French, Manda, & White, 2000; Bar et al., 1987; Wong et al., 2006) or percutaneous coronary intervention (PCI) (Armstrong et al., 2009) (de Framond et al., 2019) (Koivula et al., 2019). Clinical outcomes such as heart failure (HF) and repeat revascularization have also been consistently related to the presence of Q waves on presentation, regardless of infarct location, adequacy of ST resolution, or early presentation (Kumar et al., 2009). Recent publications have focused on the presence of Q waves or Q/R wave relation on admission ECG (Hayiroglu, Uzun, Keskin, Borklu, Tekkesin, et al., 2018); to date, however, the characteristics of these pathologic Q waves have not been subjected to analysis. Serum biomarkers such as N‐terminal pro‐b‐type natriuretic peptide (NT‐pro‐BNP) and high‐sensitivity cardiac troponin I (hs‐cTnI) have also been found to be predictors of cardiac outcomes after MI (Radosavljevic‐Radovanovic et al., 2016) (Jansen et al., 2019). However, the relationship between serum biomarkers associated with left ventricular systolic dysfunction (LVSD) or HF on the one hand and ECG parameters on the other hand is currently unexplored. In the present study, we analyze the prognostic value of specific characteristics of QRS complex and pathologic Q waves observed on the ECG of patients with anterior ST‐elevated myocardial infarction (STEMI) undergoing PCI.

## 2 METHODS

### 2.1 Background and study design

This study was performed within the framework of the BACS & BAMI Project (Biomarkers in Acute Coronary Syndrome & Biomarkers in Acute Myocardial Infarction), and all data have been obtained from the project database.

The BACS and BAMI studies included patients admitted to five hospitals in Madrid with either non‐ST‐elevation acute coronary syndrome or STEMI. Inclusion criteria have been defined previously (Tuñón, Blanco‐Colio, et al., 2014). Exclusion criteria were as follows: age over 85 years, coexistence of other significant cardiac disorders except left ventricular hypertrophy secondary to hypertension, coexistence of any illness or toxic habits that could limit patient survival, impossibility to perform revascularization when indicated, and subjects to whom follow‐up was not possible. In order to avoid variability of findings due to an excessive heterogeneity in the intervals between the acute event and blood extraction, the investigators agreed to exclude patients that were not clinically stable the sixth day after the index event. Once patients were stable, a second plasma sample was obtained between 6 and 12 months after hospital admission.

Between July 2006 and June 2014, 2,740 patients were discharged from the study hospitals with a diagnosis of non‐ST‐elevation acute coronary syndrome or STEMI. For this analysis, we selected only those patients suffering from anterior wall STEMI hospitalized in either the Fundación Jiménez Díaz Hospital or Puerta de Hierro Hospital. STEMI was defined as chest discomfort or other symptoms suggestive of ischemia lasting at least 20 min and ST‐segment elevation in at least two contiguous leads associated with a rise in myocardial injury biomarkers. Anterior STEMI was defined as ST‐segment elevation in two contiguous leads between V_2_ and V_5_. Patients with left bundle branch block were excluded from the study to avoid possible confounders in Q‐wave assessment.

### 2.2 Study protocol

Patients were included during the index admission due to an anterior STEMI event. During this hospitalization, several tests were performed following hospital's protocol, including an echocardiogram within first 12 hr after admission. Six to 12 months after the index event, a plasma sample was obtained including pro‐BNP, galectin‐3, hs‐cTnI, fibroblast growth factor 23 (FGF‐23), and parathormone (PTH). Laboratory analyses were carried out at the Clinical Biochemistry Laboratory at the IIS‐Fundación Jiménez Díaz by investigators who were unaware of the clinical data. NT‐pro‐BNP was assessed by immunoassay (VITROS; Ortho Clinical Diagnostics Raritan), and Hs‐cTnI was assessed by direct chemiluminescence (ADVIA Centaur; Siemens). FGF‐23 was measured by an enzyme‐linked immunosorbent assay that recognizes epitopes within the carboxyl‐terminal portion of FGF‐23 (Human FGF‐23, C‐Term; Immutopics Inc.), and intact PTH was analyzed by a second‐generation automated chemiluminescent method (Elecsys 2010 Platform; Roche Diagnostics). Lipid, glucose, and creatinine levels were determined by standard methods (ADVIA 2400 Chemistry System; Siemens).

An echocardiogram was also performed 6 months after the ischemic event, and ejection fraction was assessed by an expert. At the end of the follow‐up period, medical records were reviewed for cardiovascular (CV) events, and clinical status was confirmed through telephone contact.

### 2.3 Electrocardiographic analysis

We analyzed the first ECG obtained upon presentation to the hospital (admission ECG) and the last ECG before discharge (discharge ECG). All ECGs were performed in the emergency department, the coronary care unit, or the cardiology ward and were filtered at 0.05–150 Hz, 10 mm/mV, and 25 mm/s paper speed. The analysis was performed by two observers using TraceMasterVue software (Philips Electronics) with the help of digital calipers and magnification. A third observer reviewed a subgroup of ECGs to calculate interobserver variability and improve the accuracy of the study. Measures were obtained using millimeters (mm) for voltage and milliseconds (ms) for duration.

The values analyzed included heart rate, rhythm, axis, P wave, PR interval, QRS complex, pathologic Q waves, ST segment, and T waves.

Pathologic Q waves were defined following the criteria found in the latest MI consensus document, the 4th Universal Definition of Myocardial Infarction, published in 2018 (Thygesen et al., 2019), that is, any Q wave in leads V_2_–V_3_ > 0.02 s or QS complex in leads V_2_–V_3_ and Q wave ≥ 0.03 s and ≥1 mm deep or QS complex in leads I, II, aVL, aVF, or V_4_–V_6_ in any two leads of a contiguous lead grouping (I, aVL; V_1_–V_6_; II, III, aVF). The presence of R wave > 0.04 s in V_1_–V_2_ and R/S > 1 with a concordant positive T wave in the absence of a conduction defect was considered to be a sign of basal MI; as a result, such findings were not recorded as pathological as we analyzed only patients with anterior MI. Q waves not meeting these criteria were not considered for the measurements.

Any pathologic Q wave was considered for the study analysis despite its location. The characteristics of QRS complex and pathologic Q waves assessed in our study were as follows: QRS width, the sum of precordial lead voltage, precordial lead minimum voltage, the sum of Q‐wave depth, the sum of Q‐wave width, mean Q‐wave depth, mean Q‐wave width, and the number of leads with Q waves.

### 2.4 Study endpoints

The primary endpoint was the development of LVSD, defined as left ventricular ejection fraction (LVEF) ≤40%, 6 months after the index MI. The secondary endpoint was the composite of HF hospitalization or death, at the end of follow‐up.

As exploratory, we evaluate the correlation with levels of various biomarkers obtained 6 months after the event, including NT‐pro‐BNP, FGF23, galectin‐3, hs‐cTnI, and PTH.

### 2.5 Statistical analysis

Continuous variables were reported as mean ± *SD* and categorical data as numbers or percentages. Continuous variables were compared using the Student *t* test when normally distributed, and the Mann–Whitney *U* test when not normally distributed. Categorical variables were compared using the chi‐square or the Fisher exact test when the conditions required for the former were not met. For paired comparisons, Student's *t* test was used for Gaussian variables, Wilcoxon nonparametric test for variables not in Gaussian distribution, and McNemar–Broker test for categorical variables.

Logistic regression was used to determine the relationship between electrocardiographic parameters and the development of LVSD below 40% at 6 months. A linear regression analysis was performed to identify the relationship between electrocardiographic parameters and levels of different biomarkers. Finally, a Cox regression analysis was carried out in order to study the association between electrocardiographic parameters and the appearance of HF or death during follow‐up.

After performing the appropriate univariate regression analysis (linear/logistic/Cox) for each study endpoint, a stepwise multivariable regression analysis was performed to search for variables predicting the study endpoints. All variables that returned a *p*‐value <.10 after univariate regression analysis were included. Data are expressed as odds ratios (ORs) or hazard ratios when appropriate, and 95% confidence intervals (CIs) are provided. Statistical analysis was performed from the binomial distribution using the Statistical Package for Social Sciences (version 20.0, SPSS, Inc.). *P* values <.05 were considered significant for all tests.

## 3 RESULTS

We included a total of 144 patients with anterior STEMI. Baseline characteristics are listed in Table 1. Mean age was 61.3 ± 12.5 years, and 80% of patients studied were men. Thirteen patients had a previous history of acute MI (9%). At the index hospitalization, most patients were in Killip class I or class II (I 81.9%, II 14.6%, III 2.1%, and IV 1.4%). Mean LVEF performed within 12 hr from hospital admission was 43.83 ± 9.92%, and an ejection fraction of 40% or less was observed in 38.9% of patients. Ninety‐five percent of patients received primary PCI of the infarct‐related artery, and 2.1% underwent fibrinolysis. Revascularization was considered complete in 79% of the patients.

**TABLE 1:** Baseline characteristics

Sex, male (%)	116 (80.6)
Age, *X* ± *SD*	61.34 ± 12.56
Caucasian (%)	136 (94.4)
Hypertension (%)	70 (48.6)
Smoker (%)	64 (44.4)
Diabetes mellitus type 2 (%)	28 (19.4)
BMI, *X* ± *SD*	27.85 ± 4.05
Waist circumference, *X* ± *SD*	100.67 ± 11.61
Atrial fibrillation (%)	1 (0.7)
Cerebrovascular disease (%)	2 (1.4)
Peripheral artery disease (%)	5 (3.5)
Previous STEMI (%)	8 (5.6)
Previous non‐STEMI (%)	3 (2.1)
Index event	
KILLIP classification (%)	
I	118 (81.9)
II	21 (14.6)
III	3 (2.1)
IV	2 (1.4)
Heart failure during hospitalization (%)	25 (17.4)
LVEF, *X* ± *SD*	43.83 ± 9.92
LVEF ≤ 40% (%)	56 (38.9)
Analytic parameters: *X* ± *SD* (normal values)	
TnI (ng/ml)	90.1 ± 89.31 (<0.12)
CK‐MB (ng/ml)	112.47 ± 99.59 (<3.6)
CPK (UI/L)	2,046.77 ± 1,821.12 (<190)
Hemoglobin (g/dl)	15.04 ± 1.44 (12–16)
Platelets (×10^3^μl)	233.53 ± 57.48 (150–450)
Creatinine (mg/dl)	0.93 ± 0.66 (0.51–0.95)
Glucose (mg/dl)	120.32 ± 45.12 (74–109)
LDL cholesterol (mg/dl)	123.51 ± 41.13 (<160)
Ischemic times	
Time from symptom onset to first ECG (hr)	5.53 ± 12.66
System delay (hr)^a^	1.65 ± 1.85
Total ischemic time (hr)	7.04 ± 13.56
PCI LAD or branches (%)	119 (82.6)
Proximal LAD	51 (35.4)
Mid‐LAD	68 (47.2)
Distal LAD	16 (11.1)
First diagonal	20 (13.9)
Second diagonal	4 (2.8)
PCI LM (%)	2 (1.4)
Multivessel artery disease	28 (19.4)
Revascularization (%)	
No reperfusion	6 (4.2)
Primary PCI	130 (91.5)
Fibrinolysis	3 (2.1)
Fibrinolysis and rescue PCI	3 (2.1)
At least one DES implanted (%)	102 (70.8)
Complete revascularization (%)	113 (79)

Abbreviations: BMI, body mass index; CPK, creatine phosphokinase I; DES, drug‐eluting stent; LAD, left anterior descending; LDL, low‐density lipoprotein; LM, left main; LVEF, left ventricular ejection fraction; PCI, percutaneous coronary intervention; STEMI, ST‐elevated myocardial infarction; TnI, troponin.

^a^ Time from STEMI diagnosis to artery reperfusion.

Table 2 shows electrocardiographic parameters analyzed in the overall population, including admission and discharge ECG. Interobserver variability analysis revealed high accuracy with an intraclass correlation coefficient >0.75 for the most relevant electrocardiographic parameters used in subsequent analysis, indicating high similarity between values within the same group despite analysis by different observers (Table S1).

**TABLE 2:** Electrocardiographic parameters in whole study population

	Admission ECG (*X* ± *SD*)	Discharge ECG (*X* ± *SD*)
QRS width (ms)	97.71 ± 19.42	96.84 ± 18.75
Sum of the precordial lead voltage (mm)	63.24 ± 22.12	66.81 ± 23.51
Sum of Q‐wave depth (mm)	11.12 ± 12.36	12.36 ± 13.13
Mean Q‐wave depth (mm)	3.61 ± 3.12	4.08 ± 3.54
Mean Q‐wave width (ms)	44.81 ± 18.61	48.74 ± 19.64
Sum of Q‐wave width (ms)	140.15 ± 110.36	146.71 ± 119.28
Number of Q waves	2.72 ± 1.87	2.67 ± 1.84
Precordial lead minimum voltage (mm)	5.64 ± 2.69	5.89 ± 2.56
Number of leads with ST elevation	4.97 ± 1.83	3.72 ± 2

Note

1 mm = 0.1 mv.

Abbreviations: mm, millimeters; ms, milliseconds.

### 3.1 Electrocardiographic analysis and left ventricular systolic dysfunction

An ejection fraction of 40% or less was found in 20 patients (14.7%) 6 months after the index event. Patients with LVEF equal or below 40% at 6 months had a wider QRS complex and lower precordial lead minimum voltage on admission ECG. On discharge ECG, Q waves were significantly wider and deeper among those patients with LVEF ≤ 40% at 6 months. Moreover, the number of Q waves and Q‐wave depth was higher on discharge ECG of patients with LVEF ≤ 40% (Table 3).

**TABLE 3:** Electrocardiographic parameters divided into 2 groups according to LVEF at 6 months

	LVEF ≤ 40%	LVEF > 40%	*p*
Admission ECG			
Sum of the precordial lead voltage (mm)	58.26 ± 17.45	64.87 ± 23.06	.24
Sum of Q‐wave depth (mm)	14.37 ± 14.05	10.22 ± 11.92	.26
Sum of Q‐wave width (ms)	185.21 ± 159.87	124.82 ± 88.31	.13
Number of Q waves	3.26 ± 1.69	2.58 ± 1.8	.09
Mean Q‐wave depth (mm)	4.27 ± 3.62	3.49 ± 3.09	.39
Mean Q‐wave width (ms)	51.41 ± 25.47	42.86 ± 16.7	.11
Precordial lead minimum voltage	4.73 ± 2.76	5.87 ± 2.67	.**04**
QRS width (ms)	105.79 ± 25.05	94.54 ± 15.58	.**04**
Discharge ECG			
Sum of the precordial lead voltage (mm)	62.44 ± 17.24	68.94 ± 23.97	.27
Sum of Q‐wave depth (mm)	21.12 ± 17.23	10.87 ± 12.08	.**01**
Sum of Q‐wave width (ms)	216.35 ± 148.89	127.27 ± 97.59	.**01**
Number of Q waves	3.5 ± 2.33	2.42 ± 1.67	.**05**
Mean Q‐wave depth (mm)	5.31 ± 3.24	3.96 ± 3.65	.**03**
Mean Q‐wave width (ms)	54.22 ± 12.79	47.51 ± 20.66	.19
Precordial lead minimum voltage	4.94 ± 2.01	6.18 ± 2.62	.06
QRS width (ms)	102.72 ± 21.63	94.04 ± 16.12	.23

Note

1 mm = 0.1mv.

Abbreviations: mm, millimeters; ms, milliseconds.

Statistically significant values are shown in bold.

Variables related to the development of LVEF ≤ 40% at 6 months after univariate analysis are shown in Table S2. Multivariate logistic regression analysis revealed that on admission ECG, QRS width (OR 1.056 [1.022–1.052] *p* = .001) was an independent predictor of LVSD development. On discharge ECG, the sum of Q‐wave depth (OR 1.062 [1.022–1.102] *p* = .002) also resulted as independent predictor of LVEF < 40%.

### 3.2 Electrocardiographic parameters and cardiovascular events

After a median follow‐up of 2.9 ± 1.5 years, 12 patients (8.4%) developed CV events, defined as HF or death. There were eight episodes of HF requiring hospitalization and eight deaths. On univariate analysis, QRS width and mean Q‐wave width on admission ECG reached statistical significance. Similarly, the QRS width and sum of Q‐wave width on discharge ECG were significantly related to the presence of CV events (Table 4).

**TABLE 4:** Cox regression analysis of cardiac events: death or heart failure

	Univariate analysis		Multivariate analysis^a^	
	HR (95% CI)	*p*	HR (95% CI)	*p*
Admission ECG				
QRS width	1.029 (1.009–1.049)	.004	1.029 (1.009–1.049)	.004
Mean Q‐wave width	1.037 (1.003–1.073)	.033		
Sum of Q‐wave width	1.004 (1.000–1.008)	.071		
Discharge ECG				
Sum of Q‐wave width	1.006 (1.001–1.010)	.010		
QRS width	1.031 (1.004–1.059)	.026		

Abbreviations: CIs, confidence intervals; HRs, hazard ratios.

^a^ Only those parameters with statistically significant values are reported on the multivariate analysis.

On multivariate Cox regression analysis, QRS width on admission ECG was related to a 1.029‐fold increased risk of HF or death during follow‐up (Table 4).

### 3.3 Electrocardiographic parameters and biomarker analysis at 6 months

Several biomarkers were measured 6 months after index event. Mean levels of PTH were 59.69 ± 23.65 pg/ml. FGF23 levels were 100.08 (33.40–950,00)RU/ml, and galectin‐3 levels were 8.91 ± 3.95 ng/ml. NT‐pro‐BNP levels were 453.73 ± 587.51 pg/ml. Mean levels of hs‐cTnI were 9.85 ± 16.12 × 10^–3^ ng/ml.

QRS width on admission ECG was the only ECG parameter shown to be related to the higher levels of NT‐pro‐BNP at 6 months (9.24, 95% CI [2.95–15.54] *p* = .004).

Moreover, the sum of Q‐wave depth (0.35 × 10^−3^, 95% CI [0.08 × 10^−3^–0.62 × 10^−3^] *p* = .012) and the sum of Q‐wave width (0.03 × 10^−3^, 95% CI [0.01 × 10^−3^–0.07 × 10^−3^] *p* = .021) on admission ECG were related to the increased levels of hs‐cTnI at 6 months.

The sum of the voltages in precordial leads both on admission ECG (–0.246, 95% CI [–0.436–(–0.057)] *p* = .011) and discharge ECG (−0.245, 95% CI [–0.485–(–0.004)] *p* = .046) was related to the lower levels of PTH at 6 months.

No significant relationship was found between any ECG parameter and galectin‐3 and FGF‐23.

## 4 DISCUSSION

Our study underscores the importance of electrocardiography at admission and discharge in patients hospitalized for anterior STEMI as a tool for predicting clinical outcomes. We found that exhaustive analysis of QRS complexes and pathological Q waves on admission and discharge ECG could predict the presence of LVSD, the increased levels of several biomarkers at 6 months, and the development of cardiac events such as HF or death during follow‐up. The significant association between cardiac biomarkers and ECG parameters is a novel finding, as this relationship has never been explored before.

For many years, ECG has been an object of study because it is a simple, fast, and cost‐effective technique that is available in most every emergency departments. Analysis of ECG at the time of hospital admission for MI has mainly focused on the QRS complex, including the parameters of Q and R waves (Liu et al., 2020). These parameters can predict larger infarct size, poor ST‐segment recovery, and the no‐reflow phenomenon, all of which are related to worse prognosis and more negative cardiac outcomes after the MI event (Hayiroglu, Uzun, Keskin, Borklu, Turkkan, et al., 2018; Ito et al., 1996; Wong et al., 2002). Our study adds discharge ECG to the set of readings to aid in STEMI evaluation. Performed a few days after the event, ECG findings on discharge may indicate changes that will remain after the MI is established and treated. Indeed, we found the sum of the Q‐wave depth on discharge ECG showed significant value for predicting the development of LVEF ≤ 40%. Moreover, a recent substudy from the CIRCUS trial revealed that persistent Q waves after reperfusion in patients with anterior MI increased the risk of developing HF or death (de Framond et al., 2019). In our study, QRS width on admission ECG predicted the development of HF or death during follow‐up. Previous studies demonstrated that the presence of Q waves on admission during STEMI was related to worse outcomes as measured in terms of death, repeat MI, or HF compared with those patients without Q waves on their ECGs (Armstrong et al., 2009; Kumar et al., 2009). We performed a more exhaustive analysis, including the characteristics of these pathologic Q waves (width, depth), precordial lead voltages, and QRS measurement to add prognostic power to the evidence in the literature.

Cardiac biomarkers have also been the subject of study in patients with MI, not only during the acute diagnosis, but also through the subsequent months, as these biomarkers can partially predict cardiac outcomes. The higher levels of NT‐pro‐BNP values 6 months after MI were found to be a good marker of infarct size and were associated with LVSD, HF, and recurrent ischemic events during follow‐up (Kleczynski et al., 2013; Radosavljevic‐Radovanovic et al., 2016). Our results show that QRS width on the admission ECG was not only significantly correlated with higher NT‐pro‐BNP values at 6 months but also served as an independent predictor of HF and mortality during follow‐up. This finding reinforces the importance of a wider QRS to predict adverse cardiac outcomes in light of the correlation between wider QRS and increased levels of NT‐pro‐BNP 6 months after MI. Furthermore, hs‐cTnI has been shown to be related to recurrent CV events in patients with stable coronary heart disease (Jansen et al., 2019). The relationship between a wider and deeper Q wave on admission ECG and increased levels of hs‐cTnI is a novel finding of our study.

The components of mineral metabolism and phosphate levels have also been studied as predictors of CV risk. In a recent meta‐analysis of prospective trials, high PTH concentrations were associated with increased risk of CV events in general population (Kestenbaum et al., 2011; van Ballegooijen, Reinders, Visser, & Brouwer, 2013). The combination of calcidiol and FGF‐23 plasma levels has been found to be a strong predictor of adverse events in patients with CAD in a recent study. This same study also showed higher PTH levels in those patients that developed acute ischemic events (acute coronary syndromes, strokes, or transient ischemic attacks) (Tuñón, Cristóbal, et al., 2014). Our current results show an association between precordial QRS voltages on both admission and discharge ECG and PTH levels, thus revealing that as the precordial QRS voltage decreases, PTH levels determined at 6‐month increase. It is known that low QRS voltage on the presenting ECG can predict short‐ and long‐term mortality in patients with acute MI in the prethrombolytic era (Fox, Tomlinson, Meek, Portal, & Aber, 1975). In addition, it has also shown its predictive value in CV mortality in a more recent study that has reported worse LVEF and higher rates of in‐hospital and 6‐month mortality in patients with acute coronary syndrome (Tan et al., 2015). The relationship found in our study between lower QRS voltage on ECG after acute MI and higher PTH levels may mean that both parameters are markers of a worse CV prognosis.

We believe that our findings concerning ECG parameters and biomarkers (NT‐pro‐BNP, hs‐cTnI, and PTH) are novel and contribute to previous data in the literature, suggesting an added value afforded by these measures.

Our study has some limitations. First, it was an observational, two‐center study with a limited sample size, though one that was performed in high‐volume interventional hospitals where every consecutive patient who met inclusion was included, thus reducing the selection bias. Secondly, patients with a previous history of MI were not excluded from the study as has been done in previous studies to avoid bias in case the reference ECG was affected by the previous event. The number of patients in our cohort with previous STEMI was low and their respective reference ECGs were reviewed by the investigators, ruling out any modification affecting the anterior territory in the ECG. Finally, as our sample size was limited, the study findings should be taken cautiously and some of them should be considered as hypothesis generators.

The present study suggests that in patients suffering from anterior STEMI, specific electrocardiographic parameters at baseline and discharge, such as QRS width and pathological Q‐wave depth and width, may predict the development of LVSD at 6 months and the rise in several biomarkers associated with increased CV risk. QRS width on admission ECG seems to be an early predictor of HF or death after anterior wall STEMI. In summary, our findings highlight the importance of the initial ECG analysis to predict long‐term prognosis. Further investigations in larger cohorts are needed to validate these results.

## CONFLICT OF INTEREST

None.

## AUTHOR CONTRIBUTION

Marta López‐Castillo: Conception, design, acquisition of data, analysis and interpretation of data, drafting of the manuscript, final approval of the manuscript submitted. Álvaro Aceña: Conception, design, analysis and interpretation of data; revising the manuscript critically for important intellectual content; final approval of the manuscript. Ana M. Pello‐Lázaro: Conception, design, analysis and interpretation of data; revising the manuscript critically for important intellectual content; final approval of the manuscript. Vanessa Viegas: Acquisition of data, drafting of the manuscript, revising the manuscript critically for important intellectual content, final approval of the manuscript. Beatriz Merchán Muñoz: Acquisition of data, drafting of the manuscript, revising the manuscript critically for important intellectual content, final approval of the manuscript. Rocio Carda: Conception, design, analysis and interpretation of data; revising the manuscript critically for important intellectual content; final approval of the manuscript. Juan Franco‐Peláez: analysis and interpretation of data; revising the manuscript critically for important intellectual content; final approval of the manuscript submitted. Maria Luisa Martín‐Mariscal: design, acquisition of data; revising the manuscript critically for important intellectual content; final approval of the manuscript. Sem Briongos‐Figuero: Conception, design, analysis and interpretation of data; revising the manuscript critically for important intellectual content; final approval of the manuscript. Jose Tuñón: concept, design, analysis and interpretation of data; revising the manuscript critically for important intellectual content; final approval of the manuscript submitted.

## ETHICAL APPROVAL

The study was presented and approved by the ethics committee of our institution (FJD, Madrid, Spain). All patients provided signed informed consent at the moment of study inclusion.

## Supporting information

Table S1‐S2
