# Supplementary material for: Prognostic value of initial QRS analysis in anterior STEMI: Correlation with left ventricular systolic dysfunction, serum biomarkers, and cardiac outcomes
Source: Ann Noninvasive Electrocardiol. 2020 Aug 26;26(1):e12791. doi: 10.1111/anec.12791 (PMC7816810; doi:10.1111/anec.12791)
Supplement: Supplementary file 1 — Table S1‐S2 [file ANEC-26-e12791-s001.docx]

**SUPPLEMENTARY MATERIAL**

**Table 1. Inter-observer variability evaluated by Intraclass correlation coefficient (Icc)**

|  | **Icc** | **p** |
| --- | --- | --- |
| **Admission ECG** | | |
| Sum of precordial leads voltage | 0.99 | 0.001 |
| Sum of Q-wave depth | 0.97 | 0.001 |
| Mean Q-wave depth | 0.87 | 0.004 |
| Mean Q-wave width | 0.98 | 0.001 |
| Sum of Q-wave width | 0.97 | 0.001 |
| Number of Q waves | 0.83 | 0.007 |
| Precordial leads minimum voltage | 0.99 | 0.001 |
| **Discharge ECG** | | |
| Sum of precordial leads voltage | 0.85 | 0.005 |
| Sum of Q-wave depth | 0.67 | 0.069 |
| Mean Q-wave depth | 0.68 | 0.06 |
| Mean Q-wave width | 0.93 | 0.001 |
| Sum of Q-wave width | 0.98 | 0.001 |
| Number of Q waves | 0.97 | 0.001 |
| Precordial leads minimum voltage | 0.98 | 0.001 |

**Table 2. Logistic regression. Electrocardiographic parameters and development of LVSD equal or below 40% at 6 months.**

|  | Univariate analysis | | Multivariate analysis* | |
| --- | --- | --- | --- | --- |
|  | OR (IC 95%) | p | OR (IC 95%) | p |
| **Admission ECG** | | | | |
| QRS width | 1.029 (1.005-1.054) | 0.017 | 1.056 (1.022-1.052) | 0.001 |
| Mean Q-wave width | 1.037 (1.003-1.073) | 0.033 |  |  |
| Sum of Q-wave width | 1.005 (1.000-1.009) | 0.033 |  |  |
| Number of leads with ST elevation | 1.392(1.052-1.843) | 0.021 |  |  |
| **Discharge ECG** | | | | |
| Number of Q waves | 1.334 (1.037-1.718) | 0.025 |  |  |
| Sum of Q-wave width | 1.006 (1.002-1.010) | 0.005 |  |  |
| Sum of Q-wave depth | 1.048 (1.013-1.084) | 0.007 | 1.062 (1.022-1.102) | 0.002 |
| QRS width | 1.030 (1.000-1.061) | 0.047 |  |  |

*Only those parameters with statistically significant values are reported on the multivariate analysis.
